# Genome Sequence of the Versatile Fish Pathogen *Edwardsiella tarda* Provides Insights into its Adaptation to Broad Host Ranges and Intracellular Niches

**DOI:** 10.1371/journal.pone.0007646

**Published:** 2009-10-29

**Authors:** Qiyao Wang, Minjun Yang, Jingfan Xiao, Haizhen Wu, Xin Wang, Yuanzhi Lv, Lili Xu, Huajun Zheng, Shengyue Wang, Guoping Zhao, Qin Liu, Yuanxing Zhang

**Affiliations:** 1 State Key Laboratory of Bioreactor Engineering, East China University of Science and Technology, Shanghai, People's Republic of China; 2 Shanghai - MOST Key Laboratory of Health and Disease Genomics, Chinese National Human Genome Center at Shanghai, Shanghai, China; University of Hyderabad, India

## Abstract

**Background:**

*Edwardsiella tarda* is the etiologic agent of edwardsiellosis, a devastating fish disease prevailing in worldwide aquaculture industries. Here we describe the complete genome of *E. tarda*, EIB202, a highly virulent and multi-drug resistant isolate in China.

**Methodology/Principal Findings:**

*E. tarda* EIB202 possesses a single chromosome of 3,760,463 base pairs containing 3,486 predicted protein coding sequences, 8 ribosomal rRNA operons, and 95 tRNA genes, and a 43,703 bp conjugative plasmid harboring multi-drug resistant determinants and encoding type IV A secretion system components. We identified a full spectrum of genetic properties related to its genome plasticity such as repeated sequences, insertion sequences, phage-like proteins, integrases, recombinases and genomic islands. In addition, analysis also indicated that a substantial proportion of the *E. tarda* genome might be devoted to the growth and survival under diverse conditions including intracellular niches, with a large number of aerobic or anaerobic respiration-associated proteins, signal transduction proteins as well as proteins involved in various stress adaptations. A pool of genes for secretion systems, pili formation, nonfimbrial adhesions, invasions and hemagglutinins, chondroitinases, hemolysins, iron scavenging systems as well as the incomplete flagellar biogenesis might feature its surface structures and pathogenesis in a fish body.

**Conclusion/Significance:**

Genomic analysis of the bacterium offered insights into the phylogeny, metabolism, drug-resistance, stress adaptation, and virulence characteristics of this versatile pathogen, which constitutes an important first step in understanding the pathogenesis of *E. tarda* to facilitate construction of a practical effective vaccine used for combating fish edwardsiellosis.

## Introduction

*Edwardsiella tarda*, a Gram-negative bacteria belonging to *Enterobacteriaceae*, is the etiological agent for edwardsiellosis, a devastating fish disease prevailing in worldwide aquaculture industries and accounting for severe economical losses [1], [2]. The organism commonly affects more than 20 species of freshwater and marine fishes including carp, tilapia, eel, catfish, mullet, salmon, trout, turbot and flounder, causing systemic hemorrhagic septicemia and emphysematous putrefactive disease with swelling skin lesions, as well as ulcer and necrosis in internal organs such as liver, kidney, spleen, and musculature [1]. Besides piscine species, *E. tarda* also inhabits and infects a broad range of cold or warm -blooded hosts such as reptiles, amphibians, birds, mammals and even humans [2], [3], raising a concern about *E. tarda* being a significant zoonotic pathogen.

*Edwardsiellae* bacterium resides in subgroup 3 in γ-group of Proteobacteria [2] and contains 3 species, *E. tarda*, *E. hoshinae* and *E. ictaluri*, the notorious pathogen relatively strictly inhabiting and causing enteric septicemia in Channel catfish [4]. Like phylogenetically related *Enterobacteriaceae* bacteria *Salmonella* spp. [5], *E. tarda* possesses the capacity of invading epithelial cells [6], [7] and macrophages [8], and multiplies in the cells, which is implicated to be one of the critical steps in its pathogenesis by subverting the fish immune system and causing systemic hemorrhagic septicemia [9]. For the present, the scant knowledge about the genetic basis for the intracellular lifestyle and molecular pathogenesis of *E. tarda* infection has largely hindered the development of a practical effective vaccine used for combating fish edwardsiellosis. Moreover, the criticized indiscriminate long-term application of antibiotics is marginally effective [10] and raises the increasing concern of multi-drug resistant *E. tarda* strains [11], [12], leaving satisfactory control methods of the disease currently unavailable.

To unravel the genetic properties for habitat adaptation, virulence determinants, invasive nature and multi-drug resistance of *E. tarda* and to facilitate the construction of a practical effective vaccine used for combating fish edwardsiellosis, we utilized the high-throughput pyrosequencing (454 Life Sciences Corporation) together with conventional sequencing method (PCR-based sequencing on ABI3730 automated capillary electrophoresis sequencer, Applied Biosystem Inc.) to quickly determine the complete genome sequence of *E. tarda* EIB202, a chloramphenicol, tetracycline, rifampicin, and streptomycin-resistant and highly virulent strain isolated from a recent outbreak in farmed turbot in Shandong province of China [12]. *E. tarda* EIB202 has 50% lethal doses (LD_50_) of 3.8×10^3^ colony forming units (CFU) g^−1^ for swordtail fish [12], 5×10^2^ CFU g^−1^ for zebra fish, and 4.5×10^2^ CFU g^−1^ for turbot, and displays fast growth rates in a wide range of sodium chloride concentrations (0.5%–5%) as well as temperature shifts (20°C–37°C) (our unpublished data), presenting as a versatile fish pathogen. Analysis of the complete genome sequence of EIB202 revealed a number of gene hallmarks in *E. tarda* for adaptation to broad host niches and shed lights on the mechanisms underlying the intracellular colonization of the bacterium in host cells.

## Results and Discussion

### General features of the complete chromosome sequence

**Figure 1:**
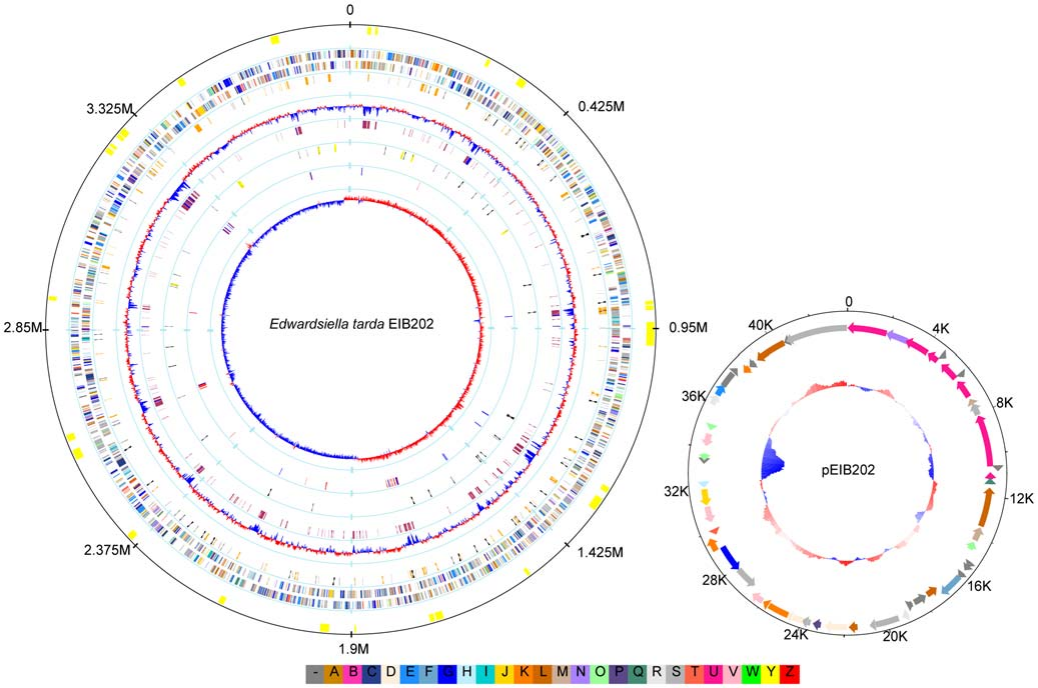
Circular atlas of *E. tarda* EIB202 genome and plasmid pEIB202. Left, chromosome; Right, plasmid. Circles range from 1 (outer circle) to 9 (inner circle) for chromosome and I (outer circle) to III (inner circle) for plasmid, respectively. Circle 1, genomic islands; Circles 2/I and 3/II, predicted coding sequences on the plus and minus strands, respectively; Circle 4, variable number of tandem repeats (VNTRs) (black) and direct repeat sequences (DRs) (orange); Circles 5/III, G+C percentage content: above median GC content (red), less than or equal to the median (blue); Circle 6, potential horizontally transferred genes and EIB202-specific genes with respect to *Enterobacteriaceae* bacteria; Circle 7, stable RNA molecules: tRNA (black), rRNA (yellow); Circle 8, phage-like genes and transposases; Circle 9, GC skew (G−C)/(G+C): values>0 (red), values<0 (blue). All genes are colored by functional categories according to COG classification: gold for translation, ribosomal structure and biogenesis; orange for RNA processing and modification; light orange for transcription; dark orange for DNA replication, recombination and repair; antique white for cell division and chromosome partitioning; pink for defense mechanisms; tomato for signal transduction mechanisms; peach for cell envelope biogenesis and outer membrane; deep pink for intracellular trafficking, secretion and vesicular transport; pale green for posttranslational modification, protein turnover and chaperones; royal blue energy production and conversion; blue for carbohydrate transport and metabolism; dodger blue for amino acid transport and metabolism; sky blue for nucleotide transport and metabolism; light blue for coenzyme metabolism; cyan for lipid metabolism; medium purple for inorganic ion transport and metabolism; aquamarine for secondary metabolites biosynthesis, transport and catabolism; gray for function unknown.

*E. tarda* EIB202 contains a single circular chromosome of 3,760,463 bp with an average G+C content of 59.7% (Table 1). The chromosome is predicted to distinctly harbor 8 rRNA operons, 95 tRNA genes, and 8 stable noncoding RNAs, relatively higher than that of other sequenced enterobacteria (Table S1) and in consistent with the rapid growth of the bacterium [12]. The eight rRNA operons, among which one operon contains a duplication in 5S rRNA gene, are scattered in the circular genome except for two locating in tandem as previously reported [13] (Figure 1). In addition to 77 pseudogenes (including 32 phage and 31 transposase genes), 3,486 coding sequences (CDSs) with an average length of 906 bp were encoded in the chromosome, representing 83.9% of the genome. Among all the protein-coding genes, 79.2% of the CDSs (n = 2,823) were assigned to a functional category of Cluster of Orthologous Groups (COG). Approximately 28% (980/3563) of the chromosomal genes are hypothetical in nature, accounting for the majority of genes (597/852) that are specific to the *E. tarda* genome among the enterobacterium genome samples.

**Table 1:** Overall features of the genome of *E. tarda* EIB202.

Chromosome	Count or percent
Size	3,760,463 bp
C+G content (%)	59.7
CDS^a^	3,486
Coding percentage	83.9%
Pseudogenes or gene fragments^b^	77
IS elements	19
rRNA genes	7*(16S+23S+5S), 1*(16S+23S+5S+5S)
tRNA genes	95
Other RNA gene	8
Average CDS length	906
**Plasmid**	
Size	43,703 bp
CDS	53
C+G content(%)	57.3
IS elements	1

Total not including pseudogenes.

Pseudogenes include transposase and phage-related genes.

### A conjugative plasmid pEIB202

A circular plasmid (designated as pEIB202) of 43,703 bp was identified from the assembled sequences. The plasmid pEIB202 carries 53 predicted CDSs, among which around 27% encode hypothetical proteins (Figure 1). The open reading frames (ORFs) of putative replication initiator protein (RepA) and plasmid partition proteins (KorA, IncC, KorB, TopA, and ParA) were found on this plasmid, suggesting that this plasmid might belong to IncP plasmid and was capable of replication and stable inheritance in a wide variety of gram-negative bacteria [14].

In the sequence of pEIB202, six genes were probably involved in resistance to antibiotics, including *tetA* and *tetR* for tetracycline, *strA* and *strB* for streptomycin, *sulII* for sulfonamide, and *catA3* for chloramphenicol resistance, providing genetic properties for previously described multi-drug resistance in EIB202 [12]. A complex transposon IS*Sf1* containing IS*4* family transposase [15] and the *catA3* gene was identified. The average G+C content of this region was observed to be comparatively extremely low (37.4%) (Figure 1), and differed by above 3σ (standard deviation) from the average G+C content of the plasmid (57.3%) or of the genome (59.7%) (σ = 0.053 for pEIB202; σ = 0.062 for the genome; window length 1.2 kb), suggesting that the chloramphenicol resistance might be recently acquired by the plasmid. Notably, the plasmid encodes an incomplete set of components involved in the type IV A secretion system (T4ASS) (*virB2*, -*B4*, -*B5*, -*B6*, -*B8*, -*B9*, -*B10*, -*B11*, -*D2*, and -*D4*). The VirB/VirD4 T4ASS was well documented in various pathogens to be involved in horizontal DNA transfer, and in secretion or injection of protein effectors into the medium milieu or into host cells [16]. In addition, several genes associated to plasmid conjugation (*mobC*, *traC*, *traD*, *traL*, *traN*, *traX*) are present in the pEIB202 sequence, demonstrating the genetic basis for its capability to transfer between bacteria in the laboratory system with a conjugation frequency of 1.6×10^−6^ (data not shown).

### Genomic plasticity and genomic islands

As illustrated by Figure 1, the G+C content of the *E. tarda* EIB202 genome is highly variable. A large portion (15%) of the genome is composed of mobile genetic elements or related to special genomic islands, displaying a mosaic structure of the genome. EIB202 contains 46 genes which are shown to be phage-like products, integrases or recombinases. In addition, a large quantity (n = 599, a total of 560 kb) of variable number of tandem repeats (VNTRs) or direct repeat sequences were detected in the genome. It also harbors 15 complete and 4 disrupted insertion sequences (IS) including 10 intact IS*100*, 2 truncated IS*100* and a copy of IS*1414I* that might lost its transposition activity as a consequence of the nonsense mutation in this insertion sequence (data not shown). Given the reported continued transposition activity of IS*100* [17], we postulated that the particular IS*100* copies were due to duplicated translocation or multiple integration events of this element occurred within this strain. Interestingly, EIB202 and *E. ictaluri* 93–146 share an insertion sequence IS*Saen1*, which could also be found in *S. enterica* serovar Enteritidis [18]. All these genes may represent tremendous potential for generating genetic diversity within protein-coding genes over a very short evolutionary time for its adaptation to various niches.

In the genome sequence of EIB202, a total of 24 genomic islands (GI) were discerned (Table 2) to scatter throughout the chromosome and contain a total of 852 EIB202-specific genes that were not found in the other *Enterobacteriaceae* bacteria investigated so far. The previously described type III secretion system (TTSS) [19] and type IV secretion system (T6SS) [19], [20] are included in the genomic islands (GI7 and GI17). The GI10 contains a mammalian Toll-like/IL-1 receptor (TIR) domain protein, a novel virulence factor implicated in the intracellular survival and lethality of *S. enteric* [21], and may also contribute to the intracellular colonization of *E. tarda* in host cells. In addition to these GIs, the regions, including GI4, GI6, GI9, and GI22 encoding type I secretion system (T1SS), hemagglutinin, O-polysaccharide (OPS) biosynthesis enzymes, and type I restriction-modification system, respectively, maybe consist of the major pathogenicity islands (PAIs) of the bacterium. Some of the GIs are flanked by tRNAs or contain transposases and prophage proteins (Table 2), indicating that these GIs are still involved in the evolution of the bacterium. Among these GIs, GI2 and GI4 are absent in the genome of *E. ictaluri* 93–146 and might be characteristics of the main difference*s* between the two species. Interestingly, all of the GIs except for GI7 and GI17, which encodes TTSS and T6SS, are absent in the phylogenetically related *Salmonella* spp., suggesting that *E. tarda*, as discussed below, the descendent of a lineage that diverged from the ancestral trunk before *Salmonella* and *Escherichia* split, might acquire these genome regions from its evolution events or *Salmonella* and its predecessors might not have acquired these GIs at the first place.

**Table 2:** Overview of the genomic islands of EIB202.

No.	CDS	Characteristics or putative functions
GI1	ETAE_0032-ETAE_0037	Peroxidase, peptidase
GI2	ETAE_0049–ETAE_0058	Hypothetical proteins
GI3	ETAE_0252–ETAE_0256	Nitrate/nitrite transporter
GI4	ETAE_0315–ETAE_0326	T1SS, invasin
GI5	ETAE_0798–ETAE_0805	Oxidoreductase, integrase
GI6	ETAE_0808–ETAE_0822	IS, hemagglutinin, haemolysin secretion system
GI7	ETAE_0839–ETAE_0892	TTSS
GI8	ETAE_1166–ETAE_1177	IS, iron uptake
GI9	ETAE_1192–ETAE_1214	OPS , CRISPR
GI10	ETAE_1390–ETAE_1396	IS, Toll-like/IL-1 receptor
GI11	ETAE_1586–ETAE_1602	IS, hypothetical proteins
GI12	ETAE_1608–ETAE_1613	Prophage, O-antigen polymerase protein
GI13	ETAE_1759–ETAE_1762	IS, acetyltransferase
GI14	ETAE_1811–ETAE_1829	Choline/carnitine/betaine transporter
GI15	ETAE_2025–ETAE_2037	Carnitine dehydratase
GI16	ETAE_2243–ETAE_2255	P-pilus related proteins
GI17	ETAE_2428–ETAE_2443	T6SS
GI18	ETAE_2465–ETAE_2476	Prophage Sf6. Flanked by tRNA-Arg
GI19	ETAE_2742–ETAE_2748	Integrase, bacteriophage proteins
GI20	ETAE_3032–ETAE_3043	Recombinase, invasin.
GI21	ETAE_3046–ETAE_3052	Transposase, chorismate mutase. Flanked by IS*100*
GI22	ETAE_3069–ETAE_3074	Type I restriction-modification system
GI23	ETAE_3078–ETAE_3091	Transposase. Flanked by tRNA-Leu and IS*100*
GI24	ETAE_3405–ETAE_3428	Transposase, phage proteins. Flanked by tRNA *selC*

### Relationship of *E. tarda* to other bacterial taxa

**Figure 2:**
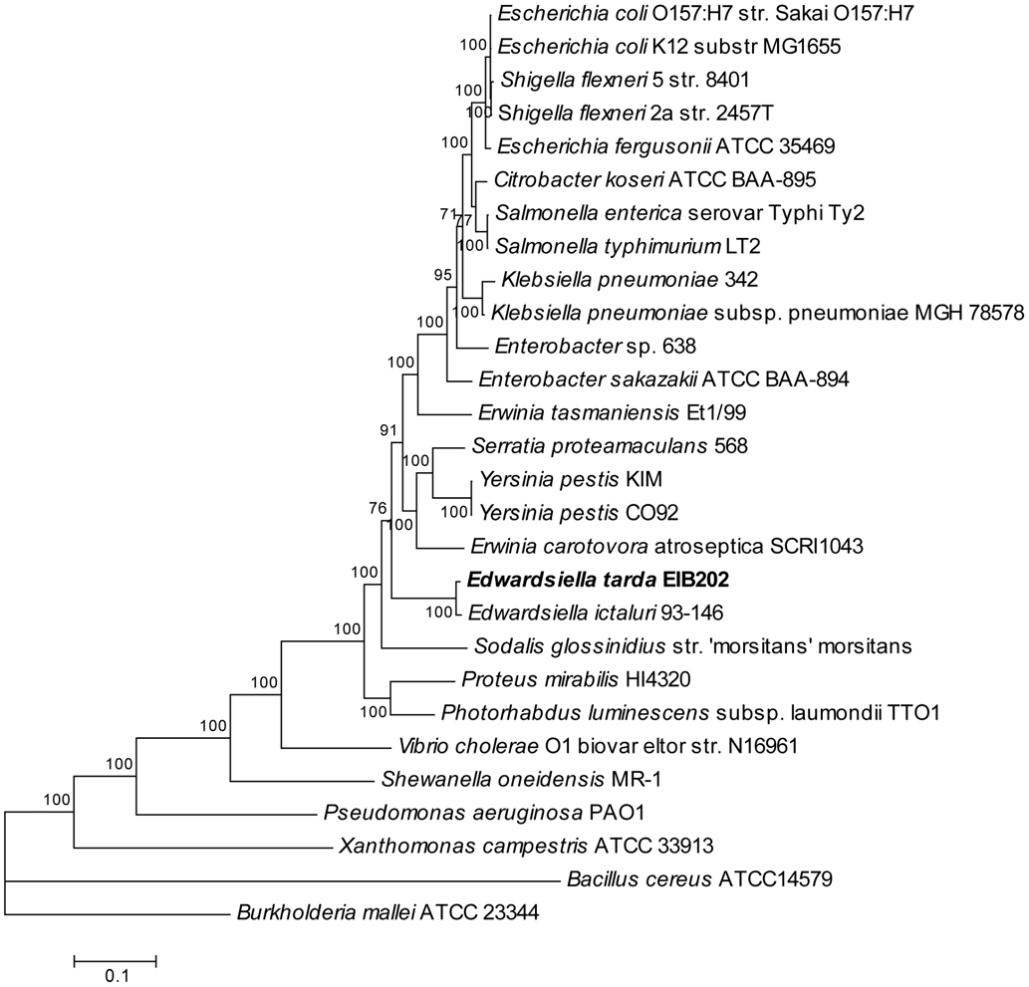
Phylogenetic relationship of EIB202. Phylogenies of *Enterobacteriaceae* species inferred from concatenated alignments of the protein sequences encoded by 44 housekeeping genes as described in the Materials and Methods. *Bacillus cereus* ATCC 14579 was used as the outgroup. Accession numbers for the selected bacterial genome sequences are as following: *Bacillus cereus* ATCC 14579, NC_004722; *Burkholderia mallei* ATCC 23344, NC_006348; *Citrobacter koseri* ATCC BAA-895, NC_009792; *E. ictaluri* 93-146, NC_012779; *E. sakazakii* ATCC BAA-894, NC_009778; *Enterobacter* sp. 638, NC_009436; *E. carotovora* atrosepticum SCRI1043, NC_004547; *E. tasmaniensis* Et1/99, NC_010694; *E. coli* K-12 substr MG1655, NC_000913; *E. coli* O157:H7 str. Sakai O157:H7, NC_002695; *E. fergusonii* ATCC 35469, NC_011740; *K. pneumoniae* 342, NC_011283; *K. pneumoniae* subsp. pneumoniae MGH 78578, NC_009648; *P. luminescens* subsp. laumondii TTO1, NC_005126; *Proteus mirabilis* HI4320, NC_010554; *Pseudomonas aeruginosa* PAO1, NC_002516; *S. enterica* subsp. enterica serovar Typhi str. Ty2, NC_004631; *S. typhimurium* LT2, NC_003197; *S. proteamaculans* 568, NC_009832; *Shewanella oneidensis* MR-1, NC_004347; *S. flexneri* 2a str. 2457T, NC_004741; *S. flexneri* 5 str. 8401, NC_008258; *Sodalis glossinidius* str. morsitans, NC_007712; *Vibrio cholera* O1 biovar eltor str. N16961, NC_002505; *Wigglesworthia brevipalpis* endosymbiont of Glossina brevipalpis, NC_004344; *Xanthomonas campestris* pv. campestris str. ATCC 33913, NC_003902; *Y. pestis* CO92, NC_003143 ; and *Y. pestis* KIM, NC_004088.

*E. tarda* shows its specific taxonomic position in *Enterobacteriaceae* as inferred from the sequence similarities of the housekeeping genes (Figure 2). At variance from the previous description that *Trabulsiella guamensis* and *Enterobacter sakazakii* were the closest relatives of *Edwardsiella* based on analysis of the limited 16S rDNA sequences [2], *E. tarda* presents as the sister clad with the phytopathogenic bacterium *Erwinia carotovora* atroseptica SCRI1043 (branch length value = 0.173), the endophytic bacterium *Serratia proteamaculans* 568 (value = 0.174), as well as human pathogen *Yersinia pestis* (value = 0.182). In addition, *E. tarda* is the most deeply diverging lineage among some notorious enteric pathogenic bacteria such as *Escherichia*, *Salmonella*, *Shigella*, and *Klebsiella*, but after the divergence of *Vibrio cholera* and *Pseudomonas aeruginosa*. The clustering of *E. tarda* EIB202 is also supported by the previous described biochemical pathways of aromatic amino acid biosynthesis and their regulation in most of the enteric bacteria [22]. Therefore, *Edwardsiella* species comprise a lineage that diverged from the ancestral trunk before the divergence of some other extensively researched enteric pathogenic bacteria, such as *Salmonella* and *Escherichia*. *E. tarda* adopts both of the intracellular and extracelluar lifestyles as its relatives such as pathogenic *S. typhimurium*, *Y. pestis* as well as symbiont *Sodalis glossinidius*, further suggesting that they experienced independent and divergent evolution driven by their specific hosts and inhabitant niches.

### Comparative genomics analysis with other enterobacteria

Utilizing the COG database [23], about 64.3% of the *E. tarda* proteins were grouped into three functional groups (Table 3), and only 14.8% were assigned to the “poorly characterized” group. The differences between *E. tarda* EIB202 and other *Enterobacteriaceae* bacteria were overviewed in Table 3. Among the whole-genome sequenced enterobacteria, *E. tarda* EIB202 contains a genome of the minimum size (Table S1), which may correspond to the previous suggestion that *E. tarda* may not be present as a free-living microorganism in natural waters but multiply intracellularly in protozoan and transmission to fish, reptile and other animals or humans [2]. Despite of the minor variations in all areas, the most obvious differences where *E. tarda* EIB202 consistently varied from all the other *Enterobacteriaceae* bacteria were discerned with the counts of *E. tarda* proteins for translation, ribosomal structure and biogenesis (J), cell envelope biogenesis, outer membrane (M), signal transduction mechanisms (T), nucleotide transport and metabolism (F), and coenzyme metabolism (H) as the highest and that for carbohydrate transport and metabolism (G) as the lowest (Table 3). The significant differences of these COG distributions were also statistically supported by the Chi-square tests using pair-wise comparisons with EIB202 (χ^2^>3.84, *P*<0.05) (Table 3). The relatively high proportion of genes in the J and M group in *E. tarda* EIB202 is consistent with the high growth rate of the bacterium as previously described [12]. Moreover, the abundance of genes in F and H group as well as the relative paucity of genes in G group may reflect that the organism is well adapted to the aquatic ecosystems and intracellular niches, where may exist relatively mean carbohydrates and wealth of nucleic acid molecules. Again, the comparatively high level of genes in signal transduction mechanisms (T) is a well manifestation of its capacities to cope with various growth conditions and to enhance its survival and persistence under a series of stresses (Table 3).

**Table 3:** Comparison of COG category distributions of EIB202 with *Enterobacteriaceae* *.

Functional categories	*E. tarda*	*S. typhimurium*	*E. carotovora*	*E. sakazakii*	*E. coli*	*K. pneumoniae*	*P. luminescens*	*S. proteamaculans*	*S. flexneri*	*Y. pestis*
**Information storage and processing**										
Translation, ribosomal structure and biogenesis (J)	171 (4.80%)	185 (3.98, 3.90%)	176 (4.95, 3.80%)	179 (3.61, 3.91%)	184 (3.03, 4.01%)	199 (5.88, 3.75%)	184 (6.95, 3.65%)	197 (4.24, 3.89%)	173 (3.51, 3.94%)	175 (2.42, 4.08%)
Transcription (K)	222 (6.23%)	329 (1.66, 6.94%)	333 (2.95, 7.19%)	290 (0.02, 6.34%)	308 (0.75, 6.71%)	445 (14.22, 8.39%)	279 (1.83, 5.54%)	449 (20.25, 8.87%)	249 (1.11, 5.67%)	237 (1.79, 5.52%)
DNA replication, recombination and repair (L)	163 (4.58%)	167 (5.89, 3.52%)	188 (1.30, 4.06%)	156 (6.90, 3.41%)	215 (0.05, 4.68%)	227 (0.45, 4.28%)	260 (1.53, 5.16%)	175 (6.96, 3.46%)	513 (127.79, 11.69%)	346 (39.02, 8.06%)
**Cellular processes**										
Cell division and chromosome partitioning (D)	34 (0.95%)	37 (0.72, 0.78%)	50 (0.31, 1.08%)	39 (0.13, 0.85%)	36 (0.68, 0.78%)	41 (0.84, 0.77%)	44 (0.15, 0.87%)	35 (1.83, 0.69%)	34 (0.75, 0.77%)	35 (0.43, 0.82%)
Defense mechanisms (V)	35 (0.98%)	49 (0.05, 1.03%)	47 (0.02, 1.02%)	43 (0.01, 0.94%)	49 (0.14, 1.07%)	74 (2.98, 1.39%)	69 (2.62, 1.37%)	57 (0.41, 1.13%)	44 (0.01, 1.00%)	43 (0.01, 1.00%)
Posttranslational modification, protein turnover, chaperones (O)	119 (3.34%)	161 (0.02, 3.40%)	140 (0.66, 3.02%)	142 (0.29, 3.11%)	138 (0.74, 3.01%)	147 (2.38, 2.77%)	118 (7.76, 2.34%)	150 (0.99, 2.96%)	131 (0.82, 2.98%)	134 (0.30, 3.12%)
Cell envelope biogenesis, outer membrane (M)	209 (5.87%)	259 (0.61, 5.47%)	240 (1.81, 5.18%)	221 (4.08, 4.83%)	239 (1.69, 5.21%)	241 (7.77, 4.54%)	203 (15.46, 4.03%)	246 (4.26, 4.86%)	213 (4.04, 4.85%)	205 (4.64, 4.78%)
Cell motility and secretion (N)	78 (2.19%)	125 (1.71, 2.64%)	116 (0.87, 2.51%)	117 (1.01, 2.56%)	116 (0.98, 2.53%)	68 (10.86, 1.28%)	105 (0.11, 2.08%)	115 (0.06, 2.27%)	89 (0.25, 2.03%)	137 (7.34, 3.19%)
Inorganic ion transport and metabolism (P)	167 (4.69%)	202 (0.86, 4.26%)	244 (1.43, 5.27%)	191 (1.13, 4.18%)	223 (0.13, 4.86%)	317 (6.83, 5.97%)	151 (16.76, 3.00%)	281 (3.15, 5.55%)	194 (0.33, 4.42%)	210 (0.18, 4.89%)
Intracellular trafficking and secretion (U)	84 (2.36%)	142 (3.13, 3.00%)	73 (6.53, 1.58%)	109 (0.00, 2.38%)	135 (2.60, 2.94%)	116 (0.29, 2.19%)	128 (0.29, 2.54%)	140 (1.37, 2.77%)	97 (0.20, 2.21%)	157 (11.06, 3.66%)
Signal transduction mechanisms (T)	151 (4.24%)	183 (0.75, 3.86%)	121 (16.56, 2.61%)	191 (0.01, 4.18%)	184 (0.27, 4.01%)	200 (1.24, 3.77%)	137 (14.89, 2.72%)	191 (1.20, 3.77%)	151 (3.44, 3.44%)	130 (8.27, 3.03%)
**Metabolism**										
Energy production and conversion (C)	214 (6.01%)	292 (0.09, 6.16%)	232 (3.88, 5.01%)	203 (9.81, 4.44%)	291 (0.38, 6.34%)	296 (0.72, 5.58%)	169 (34.52, 3.35%)	280 (0.88, 5.53%)	245 (0.66, 5.58%)	183 (12.34, 4.26%)
Carbohydrate transport and metabolism (G)	237 (6.65%)	393 (7.81, 8.29%)	353 (2.85, 7.63%)	340 (1.74, 7.43%)	377 (6.99, 8.21%)	529 (29.70, 9.97%)	189 (37.33, 3.75%)	424 (8.75, 8.37%)	319 (1.14, 7.27%)	320 (1.90, 7.46%)
Amino acid transport and metabolism (E)	291 (8.17%)	356 (1.22, 7.51%)	380 (0.00, 8.21%)	326 (2.95, 7.13%)	367 (0.08, 7.99%)	468 (1.15, 8.82%)	312 (12.50, 6.19%)	482 (4.67, 9.52%)	306 (4.07, 6.97%)	303 (3.43, 7.06%)
Nucleotide transport and metabolism (F)	89 (2.50%)	89 (3.73, 1.88%)	85 (4.25, 1.84%)	85 (3.62, 1.86%)	97 (1.34, 2.11%)	97 (4.67, 1.83%)	79 (9.43, 1.57%)	110 (0.99, 2.17%)	82 (3.72, 1.87%)	75 (5.37, 1.75%)
Coenzyme metabolism (H)	151 (4.24%)	177 (1.36, 3.74%)	131 (12.03, 2.83%)	155 (3.76, 3.39%)	155 (4.14, 3.38%)	200 (1.24, 3.77%)	188 (1.42, 3.73%)	178 (2.98, 3.52%)	151 (3.44, 3.44%)	149 (3.12, 3.47%)
Lipid metabolism (I)	75 (2.11%)	94 (0.15, 1.98%)	113 (1.01, 2.44%)	88 (0.25, 1.92%)	103 (0.18, 2.24%)	125 (0.61, 2.36%)	108 (0.01, 2.14%)	143 (4.39, 2.82%)	84 (0.37, 1.91%)	81 (0.48, 1.89%)
Secondary metabolites biosynthesis, transport and ccatabolism (Q)	44 (1.24%)	67 (0.49, 1.41%)	64 (0.34, 1.38%)	64 (0.30, 1.40%)	66 (0.62, 1.44%)	107 (7.77, 2.02%)	116 (13.02, 2.30%)	121 (14.86, 2.39%)	54 (0.00, 1.23%)	62 (0.64, 1.44%)
**Poorly characterized**										
General function prediction only (R)	285 (8.00%)	440 (9.29%)	370 (7.99%)	400 (8.75%)	401 (8.73%)	501 (9.44%)	362 (7.19%)	515 (10.17%)	362 (8.25%)	318 (7.41%)
Function unknown (S)	279 (7.83%)	374 (7.89%)	329 (7.11%)	376 (8.22%)	328 (7.14%)	390 (7.35%)	366 (7.26%)	397 (7.84%)	314 (7.15%)	369 (8.60%)

Comparison of the ORFs in each COG category (other than Chromatin structure and dynamics (B), Extracellular structures (W), Nuclear structure (Y) and Cytoskeleton (Z) due to zero values) of EIB202 and the other *Enterobacteriaceae* bacteria by using Chi-square test were described in the related text. The Chi-square value (χ^2^) and the percentage of the ORF counts in each COG category were shown within brackets. When compared with EIB202, statistically significant differences in some *Enterobacteriaceae* bacteria in each of the COG category were shown with χ^2^>3.84 (*P*<0.05).

### Predicted metabolic pathways

**Figure 3:**
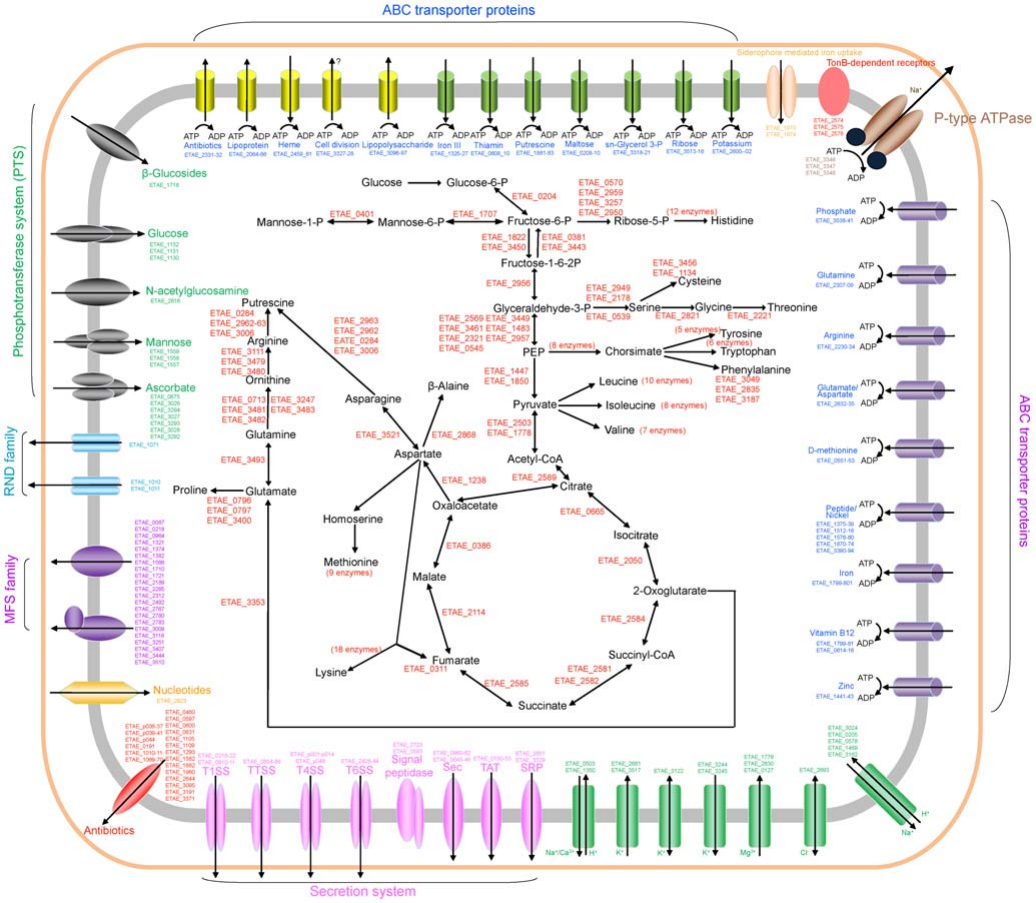
Overview of metabolism and transport in EIB202. Different transport families are distinguished by different colors and shapes. From top left going clockwise: ABC-2 and other transporters (yellow); phosphate and amino acid transporters (green); Siderophore-iron (III) receptors (brick red); TonB-dependent receptors (rosybrown); P-type ATPase (chocolate); mineral and organic ion transporters (violet); ion efflux (green); secretion systems (pink); drug/metabolite efflux (red); nucleotides transporters (orange); the major facilitator superfamily (MFS) (purple); the resistance-nodulation-cell division family (RND) (blue); phosphotransferase system (PTS) (black). Arrows indicate the direction of transport. All the amino acid biosynthesis genes are listed in Table S2.

EIB202 genome encodes the complete sets of enzymes necessary for glycolysis, the tricarboxylic cycle, the pentose phosphate pathway and Entner-Doudoroff pathway (Figure 3). In contrast, the glyoxylate shunt is not complete because isocitrate lyases (*icl1* and *icl2*) and malate synthases are missing. Except for the gene encoding pyruvate carboxylase, the complete set of genes for gluconeogenesis is present in the EIB202 genome (Figure 3). The strain also encodes a putative citrate lyase synthetase complex (ETAE_0223–0228), which may be involved in the lysis of citrate into acetate and oxaloacetate or the reverse reaction. Though genes encoding for oxalate decarboxylase, alanine transaminase and LL-diaminopimelate aminotransferase which are involved in synthesizing L-alanine were not identified in the EIB202 genome, the growth test of the bacterium indicated that it could synthesize L-alanine in an unidentified mechanism (data not shown), suggesting that the bacterium might be highly self-sufficient in amino acid biosynthesis (Figure 3 and Table S2).

EIB202 is able to produce adenosine triphosphate (ATP) through a complete respiratory chain as well as an ATP synthetase complex (ETAE_3528–3534). The genome encodes a variety of dehydrogenases (n = 80, Table 4) that enable it to draw on a variety of substrates as electron donors, such as NADH, succinate, formate, isocitrate, proline, acyl-CoA, D-amino acids and so on. The genome also encodes a number of reductases [fumarate reductase (ETAE_0335–0338), nitrate reductase (ETAE_0248–0252), dimethylsulfoxide (DMSO) reductase (ETAE_2192–2195), arsenate reductase (ETAE_1091), anaerobic sulfide reductase (ETAE_1738–1740), thiosulfate reductase (ETAE_1843–1845), anaerobic ribonucleoside triphosphate reductase (ETAE_0422–0423) and tetrathionate reductase (ETAE_1647–1649)], which may contribute to the respiration with alternative electron acceptors to oxygen (fumarate, nitrate, DMSO arsenate, thiofulfate and tetrathionate) under anaerobic conditions, which is in agreement with its facultative anaerobic lifestyle in intracellular niches.

**Table 4:** Dehydrogenases in EIB202.

CDS	Gene	Annotation
ETAE_0085	*tdh*	L-threonine 3-dehydrogenase
ETAE_0104	*wecC*	UDP-N-acetyl-D-mannosaminuronate dehydrogenase
ETAE_0240		Putative iron-containing alcohol dehydrogenase
ETAE_0386	*mdh*	Malate/lactate dehydrogenases
ETAE_0565	*thrA*	Bifunctional aspartokinase I/homeserine dehydrogenase I
ETAE_0571	*gabD*	Succinate-semialdehyde dehydrogenase I
ETAE_0587	*xdhC*	Xanthine dehydrogenase, Fe-S binding subunit
ETAE_0588	*xdhB*	Xanthine dehydrogenase, FAD-binding subunit
ETAE_0589	*xdhA*	Xanthine dehydrogenase subunit
ETAE_0601	*pdxA*	4-Hydroxythreonine-4-phosphate dehydrogenase
ETAE_0620	*leuB*	3-Isopropylmalate dehydrogenase
ETAE_0658	*pdhR*	Pyruvate dehydrogenase complex repressor
ETAE_0659	*aceE*	Pyruvate dehydrogenase subunit E1
ETAE_0662	*lpdA*	Dihydrolipoamide dehydrogenase
ETAE_0770		Putative alcohol dehydrogenase
ETAE_0899	*gldA*	Glycerol dehydrogenase
ETAE_0967	*sdr*	Short-chain dehydrogenase/reductase
ETAE_0968	*gutB*	L-iditol 2-dehydrogenase
ETAE_1048	*dld*	D-lactate dehydrogenase
ETAE_1161	*sfcA*	Malate dehydrogenase (oxaloacetate-decarboxylating)
ETAE_1202	*ugd*	UDP-glucose 6-dehydrogenase
ETAE_1212	*gnd*	6-Phosphogluconate dehydrogenase
ETAE_1248	*pyrD*	Dihydroorotate dehydrogenase 2
ETAE_1334		Iron-containing alcohol dehydrogenase
ETAE_1364		Pyruvate/2-Oxoglutarate dehydrogenase complex
ETAE_1416		D-isomer specific 2-hydroxyacid dehydrogenase NAD-binding
ETAE_1449	*zwf*	Glucose-6-phosphate 1-dehydrogenase
ETAE_1474	*dadA*	D-amino-acid dehydrogenase
ETAE_1483	*gapA*	Glyceraldehyde-3-phosphate dehydrogenase
ETAE_1508	*adhE*	Aldehyde-alcohol dehydrogenase
ETAE_1549		Short chain dehydrogenase
ETAE_1658	*putA*	Proline dehydrogenase
ETAE_1724		Short-chain alcohol dehydrogenase of unknown specificity
ETAE_1753		Short-chain dehydrogenase/reductase
ETAE_1771	*ldhA*	D-lactate dehydrogenase
ETAE_1899	*kduD*	2-Deoxy-D-gluconate 3-dehydrogenase
ETAE_1922		3-Hydroxyisobutyrate dehydrogenase and related beta-hydroxyacid dehydrogenases
ETAE_2050		Isocitrate dehydrogenase, specific for NADP+
ETAE_2070	*ndh*	NADH dehydrogenase, FAD-containing subunit
ETAE_2118		D-beta-hydroxybutyrate dehydrogenase
ETAE_2276	*hisD*	Histidinol dehydrogenase
ETAE_2380	*nuoG*	NADH dehydrogenase/NADH:ubiquinone oxidoreductase 75 kD subunit (chain G)
ETAE_2416	*asd*	Aspartate-semialdehyde dehydrogenase
ETAE_2417	*pdxB*	Erythronate-4-phosphate dehydrogenase
ETAE_2583	*sucB*	2-Oxoglutarate dehydrogenase, E2 subunit, dihydrolipoamide succinyltransferase
ETAE_2584	*sucA*	Component of the 2-oxoglutarate dehydrogenase complex,thiamin-binding
ETAE_2585	*sdhB*	Succinate dehydrogenase iron-sulfur subunit
ETAE_2586	*sdhA*	Succinate dehydrogenase catalytic subunit
ETAE_2587	*sdhD*	Succinate dehydrogenase cytochrome b556 small membrane subunit
ETAE_2588	*sdhC*	Succinate dehydrogenase cytochrome b556 large membrane subunit
ETAE_2663	*caiA*	Crotonobetainyl-CoA dehydrogenase
ETAE_2695	*folD*	Methylenetetrahydrofolate dehydrogenase (NADP(+))
ETAE_2705	*dltE*	Short chain dehydrogenase
ETAE_2787	*guaB*	Inositol-5-monophosphate dehydrogenase
ETAE_2836	*tyrA*	Bifunctional chorismate mutase/prephenate dehydrogenase
ETAE_2856	*sdhA*	Succinate dehydrogenase flavoprotein subunit
ETAE_2857	*sdhD*	Succinate dehydrogenase hydrophobic membrane anchor protein
ETAE_2858	*sdhC*	Succinate dehydrogenase cytochrome b-556 subunit
ETAE_2934	*aroE*	Shikimate 5-dehydrogenase
ETAE_2939	*gcvP*	Glycine dehydrogenase
ETAE_2949	*serA*	D-3-phosphoglycerate dehydrogenase
ETAE_2958	*epd*	D-erythrose 4-phosphate dehydrogenase
ETAE_2986		Short chain dehydrogenase
ETAE_3113	*glpC*	Sn-glycerol-3-phosphate dehydrogenase subunit C
ETAE_3114	*glpB*	Anaerobic glycerol-3-phosphate dehydrogenase subunit B
ETAE_3115	*glpA*	Sn-glycerol-3-phosphate dehydrogenase subunit A
ETAE_3141		Probable zinc-binding dehydrogenase
ETAE_3173	*xdhC*	Xanthine dehydrogenase accessory factor, putative subfamily
ETAE_3194	*aroE*	Shikimate 5-dehydrogenase
ETAE_3312	*glpD*	Glycerol-3-phosphate dehydrogenase
ETAE_3336	*fdhD*	Formate dehydrogenase accessory protein
ETAE_3337		Formate dehydrogenase-O, major subunit
ETAE_3338		Anaerobic dehydrogenases, typically selenocysteine-containing
ETAE_3339	*fdnH*	Formate dehydrogenase-N beta subunit
ETAE_3340	*fdnI*	Formate dehydrogenase-N subunit gamma
ETAE_3341	*fdhE*	Formate dehydrogenase accessory protein
ETAE_3344	*asd*	Aspartate-semialdehyde dehydrogenase
ETAE_3353	*gdhA*	Glutamate dehydrogenase/leucine dehydrogenase
ETAE_3429	*thrA*	Homoserine dehydrogenase
ETAE_3457	*gpsA*	Glycerol-3-phosphate dehydrogenase (NAD(P)+)

### Stress adaptation and signal transduction

*E. tarda* has been implicated to inhabit diverse host niches [2], where it encounters and responds to ecological changes, such as temperature change, osmolarity variation, UV/oxidative stress, pH shift, famine as well as the responsive reactions of hosts, before and during survival, invasion and cause diseases in the hosts. In *E. tarda* EIB202, an array of sigma factor (σ^70^), alternative sigma factors or extracytoplasmic function (ECF) sigma factors (σ^54^, −28, −24, −32, −38, −54) as well as anti-sigma factors were identified (Table 5), illuminating its basis to respond to various environmental or host stimuli and drive the expression of related functional genes for cellular fitness.

**Table 5:** Sigma factors and anti-simga factors in EIB202.

Type	CDS	Gene	Annotation
Sigma factor	ETAE_0454	*rpoD*	DNA-directed RNA polymerase, subunit sigma-70 RpoD
	ETAE_2728	*rpoE*	RNA polymerase factor sigma-24 RpoE
	ETAE_2126	*rpoF*	Flagellar biosynthesis factor sigma-28 FliA
	ETAE_3326	*rpoH*	RNA polymerase factor sigma-32 RpoH
	ETAE_0497	*rpoN*	RNA polymerase factor sigma-54 RpoN
	ETAE_2873	*rpoS*	RNA polymerase factor sigma-38 RpoS
Anti-sigma factor	ETAE_0508		Putative anti-sigma B factor antagonist
	ETAE_0576	*dnaK*	Molecular chaperone
	ETAE_1223	*flgM*	Anti-sigma 28 factor
	ETAE_1867	*pspA*	Phage shock protein A, suppresses σ^54^-dependent transcription
	ETAE_2684		Anti-sigma regulatory factor (Ser/Thr protein kinase)

The organism is well equipped to cope with the first main obstacle, temperature fluctuations, in aquatic ecosystems. Six homologues of cold shock proteins (CspA, −B, C, D, G, H, I), among which two copies of *cspC* were included, were discerned to represent one of the largest paralogue gene family in EIB202. Closer investigation indicated that the established cold adaptation-related proteins RpoE (ETAE_2728), RseA (ETAE_2727), Rnr (ETAE_0360), DeaD (ETAE_0411), RbfA (ETAT_0406), NusA (ETAE_0404), and PNP (ETAE_0409) were all encoded in the EIB202 genome, consisting a reservoir to cope with the physically extreme cold in the environment, and may help the organism to persist in a previously described dormant state known as viable but not culturable state (VBNC) [24]. In line with the versatility in coping with the cold scenarios, EIB202 genome also has an arsenal of 34 shock proteins (GroEL, GroES, IbpAB, GrpE, etc.) or chaperons for other environmental or host changes (Table 6). One operon, *sspAB*, as well as another conserved ORF (ETAE_2419) which was known to play essential roles in acid tolerance response, were found in the chromosome, as was the operon *pspFABCD*, *pspE* and *pspG* genes known as encoding phage shock proteins responding to various membrane stimuli other than phage induction.

**Table 6:** Shock proteins in EIB202.

Shock protein	Gene	Function
ETAE_0008	*ibpA*	Molecular chaperone (small heat shock protein)
ETAE_0009	*ibpB*	Molecular chaperone (small heat shock protein)
ETAE_0086		Putative ATPase with chaperone activity
ETAE_0235	*pspG*	Phage shock protein G
ETAE_0297	*torD*	Chaperone protein
ETAE_0313	*groES*	Co-chaperonin GroES (HSP10)
ETAE_0314	*groEL*	Chaperonin GroEL (HSP60 family)
ETAE_0576	*dnaK*	Molecular chaperone
ETAE_0577	*dnaJ*	DnaJ-class molecular chaperone with C-terminal Zn finger domain
ETAE_0746	*ompH*	Periplasmic chaperone
ETAE_0867	*escB*	Type III secretion system chaperone protein B
ETAE_0871	*escA*	Type III secretion low calcium response chaperone
ETAE_0945	*yegD*	Putative chaperone
ETAE_1031		Fimbrial chaperon protein
ETAE_1420	*torD*	Cytoplasmic chaperone TorD family protein
ETAE_1657	*htpX*	Heat shock protein
ETAE_1774	*hslJ*	Heat shock protein HslJ
ETAE_1864	*pspD*	Phage shock protein D
ETAE_1865	*pspB*	Phage shock protein B
ETAE_1867	*pspA*	Phage shock protein A (IM30), suppresses sigma54-dependent transcription
ETAE_1868	*pspF*	Phage shock protein operon transcriptional activator
ETAE_2048	*hchA*	Molecular chaperone
ETAE_2146	*fliJ*	Flagellar biosynthesis chaperone
ETAE_2218	*fimC*	Periplasmic chaperone
ETAE_2251	*papD*	Chaperone protein
ETAE_2362		Hydrogenase 2-specific chaperone
ETAE_2419		Acid shock protein precursor
ETAE_2466		Phage tail assembly chaperone gp38
ETAE_2735	*grpE*	Heat shock protein
ETAE_2811	*hscA*	Chaperone protein
ETAE_2812	*hscB*	Co-chaperone Hsc20
ETAE_2829	*clpB*	Protein disaggregation chaperone
ETAE_3271		Heat shock protein 15
ETAE_3272		Disulfide bond chaperones of the HSP33 family

Regarding to osmotic stress, EIB202 has developed the ability to tolerate high concentrations of sodium chloride (up to 5%) [12]. The genes responsible for the synthesis and uptake of several compatible solutes (osmolytes), such as *ectABC* for ectoine biosynthesis, *bccT* and *betABI* for the transport of betaine, as well as *proVWX* for the uptake and transport of proline and glycine betaine, which often reside on the genome of halophilic bacteria, are absent in EIB202. In contrast, the *caiTABCDC* (ETAE_2658–2664) and *caiF* (ETAE_2672) genes involved in carnitine/betaine uptake and metabolism are present in the genome. Carnitine is a ubiquitous substance in eukaryotes and, in coupling with its metabolic intermediates crotononbetaine and γ-butyrobetaine, may be served as osmoprotectant and stimulate growth in anaerobic and starvation conditions. Spermidine and putrescine uptake system *potABCD* (ETAE_1881–1886) and biosynthesis related genes (ETAE_2962–2963) (Figure 3) are also present in the genome which may be involved in acid resistance and biofilm formation and can act as a free radical ion scavenger [25]. The lacking of genes encoding osmolarity responding proteins OmpC and OmpF also supports the idea that EIB202 might utilize unusual mechanisms to cope with the osmosis challenges.

Two-component signal transduction system (TCS), comprising of a sensor histidine kinase (HK) protein and a response regulator (RR) protein, is well documented to regulate various biophysical processes as well as virulence in bacteria [26]. EIB202 harbors dozens of TCSs including 31 HK genes and 33 RR genes (including ETAE_1502 as an orphan RR protein) (Table 7). Most of the HK genes reside adjacent to RR genes on the chromosome and are likely to be functional pairs involved in responses to environmental changes. However, the order of these gene pairs (5′-HK/3′-RR or 5′-RR/3′-HK) and the transcriptional direction relative to the chromosome (direct or complementary) appear to be random. In this respect, four particular pairs (i.e., ArcB/ArcA, CheA/CheB/CheY, BarA/UvrY and YehU/YehT) are exceptional in the sense that each corresponding partner resides at a different location of the chromosome, although each pair is known to function together in a certain signaling pathway. In *E. tarda*, TCSs may mediate adaptive responses to a broad range of environmental stimuli, including phosphate/Mg^2+^ limitation (Pho), anaerobic condition (Cit and Arc), heavy metal overload (Cus), osmosis change (EnvZ/OmpR), and motility/chemotaxis (Che), etc. It is probably safe to conclude that most two-component regulation is used for enhancing the versatility of the response of the organism to environmental stimuli by the regulation of normally unexpressed genes, while some TCSs, such as the previously described EsrA/EsrB [19] and PhoP/PhoQ [20], may also contribute to the virulence in EIB202.

**Table 7:** Two component signal transduction system in EIB202.

Histidine protein kinase (HK)	Response regulator (RR)	HK gene	RR gene	Putative functions
ETAE_0228	ETAE_0229	*citA*	*citB*	Citrate uptake and metabolism
ETAE_0302	ETAE_0300	*torS*	*torR*	Trimethylamine N-oxide respiration
ETAE_0393	ETAE_0394	*basS*	*basR*	Modification of lipopolysaccharide
ETAE_0447	ETAE_0448	*qseC*	*qseB*	Flagellar biogenesis and motility
ETAE_0775	ETAE_0777	*yfhK*	*yfhA*	Unknown function
ETAE_0885	ETAE_0886	*esrA*	*esrB*	Protein secretion and virulence
ETAE_1068	ETAE_1067	*baeS*	*baeR*	Multidrug efflux
ETAE_1081	ETAE_1080	*phoR*	*phoB*	Phosphate limitation
ETAE_1112	ETAE_1111	*narQ*	*narP*	Nitrogen metabolism
ETAE_1319	ETAE_1318	*pgtB*	*pgtA*	Phosphoglycerate transport
ETAE_1329	ETAE_1330	*uhpB*	*uhpA*	Hexose phosphate transport
ETAE_1646	ETAE_1645			Unknown function
ETAE_1662	ETAE_1663	*cusS*	*cusR*	Heavy metal efflux
ETAE_1755	ETAE_1754	*rstB*	*rstA*	Unknown stress
ETAE_1912	ETAE_1913			Unknown function
ETAE_2010	ETAE_2011	*atoS*	*atoC*	Short chain fatty acid metabolism
ETAE_2060	ETAE_2059	*phoQ*	*phoP*	Mg^2+^ starvation and virulence
ETAE_2333/2335	ETAE_2334	*yojN/rcsC*	*rcsB*	Unknown function
ETAE_2603	ETAE_2604	*kdpD*	*kdpE*	Potassium transport
ETAE_2684	ETAE_2686		*rsbU*	Unknown function
ETAE_3010	ETAE_3009	*lytS*		Autolysis regulation
ETAE_3278	ETAE_3279	*envZ*	*ompR*	Osmosis regulation
ETAE_3354	ETAE_3355			Unknown function
ETAE_3397	ETAE_3398	*uhpB*	*uhpA*	Hexose phosphate uptake
ETAE_3454	ETAE_3453	*cpxA*	*cpxR*	Cell envelop protein folding and degradation
ETAE_3494	ETAE_3495	*ntrB*	*ntrC*	Nitrogen assimilation
ETAE_0529		*arcB*		Anaerobic respiration
	ETAE_0561		*arcA*	
	ETAE_0672		*creB*	
ETAE_1340		*cheA*		Bacterial chemotaxis
	ETAE_1346		*cheB*	
	ETAE_1347		*cheY*	
ETAE_2717		*barA*		Carbon storage regulation
	ETAE_2045		*uvrY*	
ETAE_3345		*yehU*		Unknown function
	ETAE_2838		*yehT*	
	ETAE_1502			Unknown function, orphan RR protein

Quorum sensing (QS) is the signal transduction system that responds to cell density for inter- and intra- species communication under various conditions or stresses [27]. *E. tarda* EIB202 carries AI-1/AHL dependent EdwI/EdwR (ETAE_2593/2594) system [28], AI-2/LuxS (ETAE_2854) system [29] and a putative AI-3/epinephrine/norepinephrine system which might be sensed by the QseB/QseC (ETAE_0447/0448) TCS system to activate the expression of flagellar operons and virulence related genes, the way as that in *E. coli* [30]. Also, EIB202 contains dozens of proteins that may be involved in the c-di-GMP mediated signal transduction system, including the effector protein with a PilZ domain (ETAE_3384), and 10 proteins with c-di-GMP biosynthesis related GGDEF or degradation associated EAL domain (Table 8). It is intriguing to detect another 5 proteins carrying both GGDEF and EAL domain (Table 8). These proteins may be involved in the signal transduction networks controlling the “make and break” of the second messenger c-di-GMP, which in turn binds to an unprecedented range of effector components and controls diverse targets necessary for virulence and different bacterial lifestyles in various niches [31]. In all, the abundant repertoire (5% of all the EIB202 CDS) of signal transduction related genes in terms of their numbers and families (Table 3) fundamentally contribute to the survival of EIB202 in various hosts.

**Table 8:** Proteins involved in the c-di-GMP mediated signal transduction system in EIB202.

CDS	EAL*	GGDEF*	PilZ*	Gene	Annotation
ETAE_0946	Y	Y			Hypothetical protein
ETAE_0950	Y	Y			Diguanylate cyclase/phosphodiesterase
ETAE_1159	Y	Y			Hypothetical protein
ETAE_1561	Y				Hypothetical protein
ETAE_1829	Y				Hypothetical protein
ETAE_1905		Y			Diguanylate cyclase
ETAE_1913		Y			Response regulator receiver modulated diguanylate cyclase
ETAE_2054		Y			Hypothetical protein
ETAE_2294	Y				hypothetical protein
ETAE_2320		Y			Hypothetical protein
ETAE_2699		Y		*ycdT*	Diguanylate cyclase
ETAE_2759		Y			Hypothetical protein
ETAE_3138	Y	Y		*csrD*	Regulatory protein
ETAE_3375	Y				Hypothetical protein
ETAE_3380	Y	Y			Diguanylate cyclase/phosphodiesterase
ETAE_3384			Y	*bcsA*	Cellulose synthase catalytic subunit

Y indicates presence of EAL, GGDEF or PilZ domain in the listed proteins.

### Surface structures and putative virulence factors

Previous studies have determined that *E. tarda* infects fish via the following three principle entry sites: skin, gill and intestine [6]. A variety of surface structures mediating motility, adherence and pathogen-host recognition seem to be the most important properties for the initiation of infection process in *E. tarda*. The gene clusters for P pilus (*pap* genes), type 1 fimbriae (*fim* genes) as well as several genes for other nonfimbrial adhesins, invasins and hemagglutinins (Table 9) are present in the EIB202 genome, suggesting its ability to bind to specific receptors distributed in its various hosts and therefore defining the site of entry and colonization. Interestingly, dozens of these surface structure related proteins are encoded in the EIB202 GIs such as GI4 (invasion), GI6 (hemagglutinin), GI9 (OPS biosynthesis cluster), GI12 (OPS biosynthesis protein) and GI16 (P pilus related proteins) (Table 2), further suggesting that its surface structures might be shaped by the evolution events to acquire colonization and fitness when approaching various hosts. These observations also underlie the previous descriptions regarding the various mannose-resistant hemagglutination (MRHA) and mannose-sensitive hemagglutination (MSHA) phenotypes as well as serotypes in *E. tarda* strains [2], [32], [33].

**Table 9:** Partial of surface structures and virulence related genes in EIB202.

CDS	Characteristics
ETAE_0315	Hypothetical protein, putative BAP type adhesins
ETAE_0323	Putative invasin, shdA, non-fimbrial adhesin
ETAE_0613	Putative hemolysin secretion ATP-binding protein
ETAE_0817	Filamentous haemagglutinin family outer membrane protein
ETAE_0818	Putative adhesin/hemagglutinin/hemolysin
ETAE_0821	ShlB/FhaC/HecB family haemolysin secretion/activation protein
ETAE_0910	Hemolysin transporter protein
ETAE_0911	Putative hemolysin precursor
ETAE_1008	Hemolysin expression modulating family protein
ETAE_1267	OmpA, outer membrane protein A
ETAE_1528	OmpW, outer membrane protein W
ETAE_2089	Pic serine protease precursor, FhaB filamentous heamagglutinin
ETAE_2842	Putative adhesin
ETAE_2937	Hemolysin III family
ETAE_3034	Putative invasin
ETAE_3045	Temperature sensitive hemagglutinin

**Figure 4:**
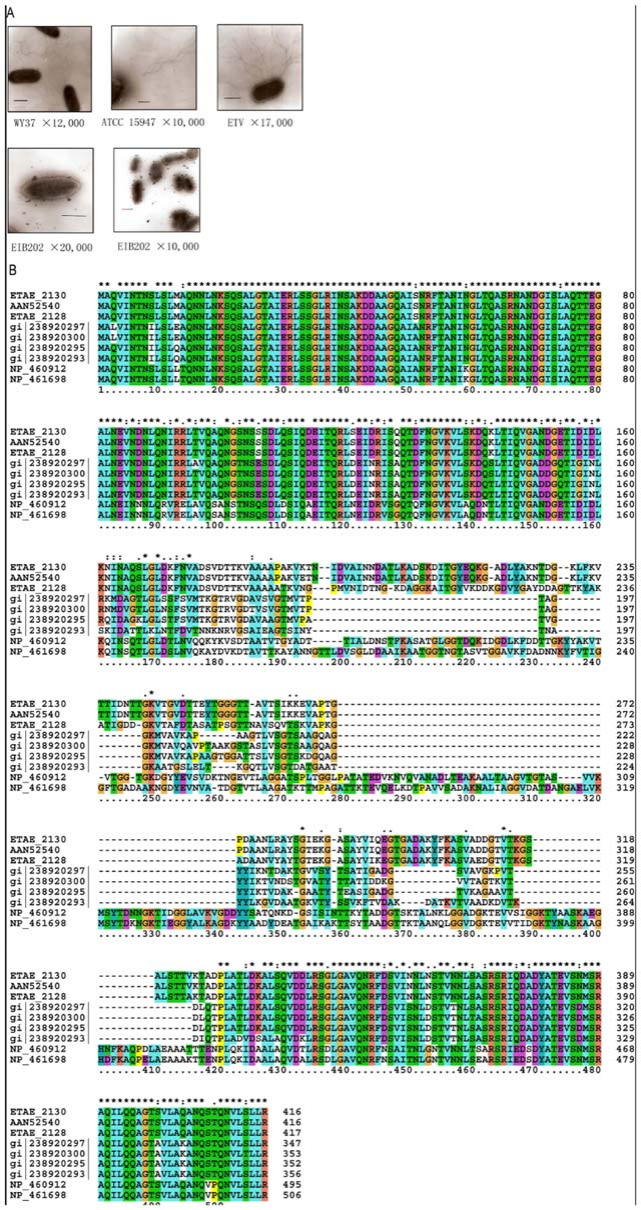
Flagella and flagellin genes of *Edwardsiella* strains. A. *E. tarda* strains WY37 (isolated from turbot), ATCC15947 (isolated from human feces) and ETV (isolated from human) were overnight cultured on LB liquid medium. Cells were collected by centrifugation at 1,000 rpm for 2 min following removing supernatant and then fixed with 2.5% glutaraldehyde. Scale bars represent 1 µm. B. The aligned putative flagellin sequences from *E. tard*a EIB202 (ETAE_2128, ETAE_2130), *E. tard*a PPD130/91 (AAN52540), *E. ictaluri* str. 93-146 (gi|238920295|, gi|238920297|, gi|238920300|) and *S. typhimurium* LT2 (NP_461698, NP_460912). Protein sequences are typically highly conserved at their C-terminal and N-terminal ends encoding the flagellar filament backbone while the middle region is generally quite variable, representing the surface-exposed and antigenically variable portion of the filament.

EIB202 was observed to be of non-motile and deficient in flagellar biosynthesis (Figure 4A). A set of early, middle and late flagellar genes displaying high similarities to *S. enterica* Serovar Typhimurium were found to be scattered present in the EIB202 genome sequence, encoding components required for flagellar hook basal body and hook-filament junction structures [34]. The main dissimilarities between the two organisms seem to lie in the late stage genes for flagella assembly. In EIB202, though two homologues of *S. enterica* phase-1 flagellin *fliC* gene were identified (ETAE_2128 and ETAE_2130) (Figure 4B), genes for *S. enterica* phase-2 flagellin (*fljB*), flagellin methylase (*fliB*), flagellin repressor (*fljA*), and methyl accepting chemotaxis component (*aer*) were absent in the EIB202 genome, which might account for the incapacity of flagellar biogenesis and weak motility in EIB202. However, the inability of flagellar biogenesis may enhance its invasion capacity by avoiding the pro-inflammatory responses and escaping the recognition by Toll-like receptor 5 [35] and the attack by caspase-1 and interleukin 1β secreted by host cells which recognize the flagellin of the bacteria mounting the host cells [36].

Surface-exposed and secreted proteins are of significance for the niche adaptations and pathogenesis of pathogens. There are various secretion pathways generally including type I to VI secretion systems and other specific protein transduction systems to fulfill the functions of protein secretion in Gram negative bacteria [37]. The Sec-dependent transport system, the components of the main terminal branch of the general secretory pathway (GSP), the signal recognition particle (SRP) and the Sec-independent twin arginine transport (Tat), T1SS, TTSS and T6SS were all identified in the genome of *E. tarda* EIB202 (Figure 3). Tat mediated protein translocation, which allows for the secretion of folded proteins such as redox proteins and their chaperones, has been demonstrated to mediate various stress responses and virulence in pathogenic bacteria, and is prevalent among halophiles and helps in transport of proteins in their folded state [38], [39]. In EIB202, a total of 33 CDSs harboring the Tat specific N-terminus signal peptide were identified to be the potential substrates of the secretion system (Table S3). These proteins, in combination with their co-translocated substrates, might also contribute to its adaptation to high salt conditions (up to 5%) and other environmental stresses. Unlike *Vibrio* spp. or other pathogens, EIB202 contains just one copy of TTSS and T6SS, and the components and genetic spatial organizations of the two types of secretion systems in EIB202 were of the same with that described in *E. tarda* strain PPD130/91 [19], [20]. The various secretion systems in EIB202 may be also involved in the transmembrane transduction of fimbrial or non-fimbrial adhesins/invasions/haemagglutinins to enrich the surface structures of the bacterium.

Survival inside the mucus and fish body seems to be attributed to a vast arsenal of proteins active against the fish tissues, epithelial/endothelial cells, fragile cells and vascular system. In spite of previous description as a mean producer of extracellular proteases [32], [33], EIB202 harbors the genes (n = 22) that encode putative extracellular proteins which may take part in the utilization of extracellular matrix such as nucleotides, and phospholipids for survival and fitness (Table 10). The genes ETAE_2918 and ETAE_2529 encoding putative chondroitinases are present in the genome and may be involved in the formation of the chronic “hole-in-the-head” lesions due to cartilage degradation [40]. With the exception of *hlyA* encoding β-hemolysin, 6 hemolysins and related function genes are found in the genome, possibly accounting for the usually observed red skin or hemorrhage symptoms in affected fish [2]. The gene encoding collagenase (ETAE_0416) may mediate vascular injury and hemorrhage symptoms during infection. Interestingly, EIB202 genome encodes a protein carrying homology to von Willebrand factor type A domain of humans (ETAE_3520, *vwA*), which is involved in the normal hemostasis by adhering to the subendothelial matrix following vascular damage [41].

**Table 10:** Putative extracellular proteins involved in matrix utilization in EIB202.

CDS	Gene	Function	Location
ETAE_0025	*uraA*	Putative xanthine/uracil permeases	Cytoplasmic/Membrane
ETAE_0130	*pldA*	Outer membrane phospholipase A	Outer membrane
ETAE_0280	*endA*	Deoxyribonuclease I	Periplasmic
ETAE_0399	*hflB*	ATP-dependent metalloprotease	Cytoplasmic/Membrane
ETAE_0511	*degS*	Serine endoprotease	Periplasmic
ETAE_0512	*degQ*	Trypsin-like serine proteases	Periplasmic
ETAE_0709	*ptrA*	Protease III precursor	Periplasmic
ETAE_0744		Putative membrane-associated zinc metalloprotease	Cytoplasmic/Membrane
ETAE_0950		Diguanylate cyclase/phosphodiesterase	Cytoplasmic/Membrane
ETAE_1034	*ushA*	5′-Nucleotidase/UDP-sugar diphosphatase	Periplasmic
ETAE_1078	*sbcC*	Nucleotide exonuclease	Extracellular
ETAE_1088	*uraA*	Xanthine/uracil permeases	Cytoplasmic/Membrane
ETAE_1747	*pntB*	Pyridine nucleotide transhydrogenase	Cytoplasmic/Membrane
ETAE_2596	*pde*	3′,5′-Cyclic-nucleotide phosphodiesterase	Periplasmic
ETAE_2704	*tesA*	Acyl-CoA thioesterase I	Periplasmic
ETAE_2771		Serine endoprotease	Periplasmic
ETAE_2918		Hypothetical chondroitinase	Outer membrane
ETAE_3117	*glpQ*	Glycerophosphoryl diester phosphodiesterase	Periplasmic
ETAE_3314	*glpG*	Predicted intramembrane serine protease	Cytoplasmic/Membrane
ETAE_3343		Putative lysophospholipase	Cytoplasmic/Membrane
ETAE_3490	*rbn*	Ribonuclease BN	Cytoplasmic/Membrane
ETAE_3545		Xanthine/uracil permeases family protein	Cytoplasmic/Membrane

*E. tarda* has developed abilities to utilize hemin, hemoglobin and hematin as iron source as well as siderophore-mediated iron uptake mechanism [2]. The finding of the clustered genes (ETAE_1793-ETAE_1801) which encode a coproporphyrinogen III oxidase (*hemN*), heme iron utilization protein, hemin receptor and related ABC transporter proteins underlies its capacities to use hemin related iron sources (Figure 3) [2]. It is interesting to discern a gene cluster (ETAE_1968-ETAE_1974) sharing high similarities to the *pvsABCDE-psuA-pvuA* operon (Figure 3) which encodes the proteins for the synthesis and utilization of vibrioferrin, an unusual type of siderophore requiring nonribosomal peptide synthetase (NRPS) independent synthetases (NIS) and usually mediating the iron uptake systems in *V. parahaemolyticus* and *V. alginolyticu*, normal marine flora as well as opportunistic pathogens for sea animals and humans [42], [43]. In EIB202 genome, genes for ferric uptake regulator, ferric reductase, ferrous iron utilization, ferritin protein and TonB systems are also present (Figure 3), implicating an elaborate iron homeostasis system priming for the inhabitation and invasion of various hosts.

### Gene properties for intracellular colonization

It has been demonstrated that EIB202 and other virulent isolates of *E. tarda* are capable of living and persisting inside the phagocytes before leading a systemic infection [44]. In the facultative intracellular pathogens such as *Mycobacterium tuberculosis* and *Salmonella*, fatty acids metabolism and the glyoxylate shunt play important roles in their long-term persistence and infection in hosts [45]. In contrast, as above-mentioned, the genes *fadD*, *fadF*, *fadE* and *icl*, which are required for fatty acids metabolism and the glyoxylate shunt, are absent in the genome sequence of EIB202, indicating that the bacterium might adopt an unusual intracellular persistent strategy to fulfill colonization and infection in various hosts.

In *E. tarda*, the ability to produce enzymes including catalase, peroxidase and superoxide dismutase (SOD) to detoxify various reactive oxygen species (ROS) has been implicated to be essential for counteracting phagocyte-mediated killing. The EIB202 genome contains genes putatively for a copper-zinc SOD (ETAE_0247) and an iron-cofactored SOD (ETAE_1676), as well as catalases (ETAE_0889 and ETAE_1368), which were believed to be the genetic marker for the *E. tarda* virulent strains [46]. Moreover, several genes (n = 9) encoding functions for protecting the cells from ROS damages with peroxidase activities (7) or repairing functions (2) are found, intriguingly including a non-haem peroxidase AhpC (ETAE_0956) for alkyl hydroperoxide reductase and a Dyp-type haem-dependent peroxidase (ETAE_1129) (Table 11). These genes confer the organism broader capacities to cope with the oxidative stresses and may necessarily contribute to the abilities to multiply inside the host cells (e.g. macrophage cells), and further the virulence of the bacterium. Actually, when the alternative sigma factor RpoS is deleted to significantly decrease the expression of SOD and catalase, the bacterium shows deficiency in the internalization and colonization of fish cells [47].

**Table 11:** ROS related proteins in EIB202.

CDS	gene	Function
ETAE_0034		Putative iron-dependent peroxidase
ETAE_0099	*trxA*	Thioredoxin domain-containing protein
ETAE_0247	*sodC*	Copper-zinc superoxide dismutase
ETAE_0889	*katG*	Putative catalase/peroxidase
ETAE_0956	*ahpC*	Alkyl hydroperoxide reductase, small subunit
ETAE_1094	*bcp*	Thioredoxin-dependent thiol peroxidase
ETAE_1129		Dyp-type peroxidase family
ETAE_1368	*katE*	Putative catalase B
ETAE_1484	*msrB*	Methionine-R-sulfoxide reductase
ETAE_1496	*xthA*	Exonuclease III
ETAE_1676	*sodB*	Superoxide dismutase
ETAE_1715	*yhjA*	Probable cytochrome C peroxidase
ETAE_1859	*tpx*	Thiol peroxidase

In *E. tarda*, the TTSS and T6SS have been demonstrated to be essential for resistance of phagocytic killing and replicating within the cells, thus important for the full virulence of the organism [19], [48], [49]. The TTSS and T6SS have been suggested to be the genetic hallmarks for the differentiation of virulent and avirulent strains of *E. tarda* [19], [20], [50]. The preservation of intact genomic islands for TTSS and T6SS in EIB202 genome will definitely potentiate it to live an intracellular life after invading hosts. As the circumstances in *Salmonella* [51], [52], the DnaK/DnaJ chaperone machinery and the type I restriction-modification system in GI22 in EIB202 may also contribute to its invasion and survival within macrophages and avoid perturbations from host immune cells for a cosy intracellular life. Interestingly, EIB202 genome harbors two separated genes, *mgtB* (ETAE_3346) and *mgtC* (ETAE_1776). Their *Salmonella* homologues are located on the *selC* locus as a *mgtCB* operon and are required for its survival within macrophages and growth in low Mg^2+^ environment [53], while the *selC* locus on EIB202 is flanked by GI24 containing prophage/transposase/integrase genes. These genetic properties provided strong evidence that EIB202 possesses the capacity of invading macrophages and subverting the fish immune systems, maybe in a manner different from that of extensively studied *Salmonella*. Further experiments are required to unravel their exact roles in the edwardsiellosis pathogenesis.

### Conclusions

*E. tarda* is well established to be one of the leading fish pathogens haunting the aquaculture industries throughout the world, and its association with high value fish species such as turbot has impelled the attempts for vaccine development against this organism. In this study, we have determined the complete genome sequence of EIB202, a highly virulent and multi-drug resistant isolate. The comprehensive analysis of the genome sequence provides evidences that the bacterium harbors an array of antibiotics-resistance determinants and well prepares for the antibiotics cocktail that might be present in the aquaculture ecosystem, similarly to that described in another *E. tarda* strain TX01 isolated from moribund turbot (*Scophthalmus maximus*) in Shandong, China [11]. The self-transmissibility of the plasmid pEIB202 further intensifies the concern that the genome contents of *E. tarda* are partly shaped by its life in various aquatic ecological niches. The findings of stress responding genes as well as signal transduction systems also confirmed the jack of all trade nature of the bacterium which could survive in a variety of hosts and growth conditions, including intracellular niches. Moreover, analysis of the genome sequence also revealed a virulence arsenal in the bacterium, confirming special pathogenic mechanisms of the organism. The determination of genome sequence of the bacterium will undoubtedly facilitate our understanding of this organism and will set the basis for vaccine development using the “reverse genetics” approach.

## Materials and Methods

### Bacterial growth and DNA extraction

*E. tarda* EIB202 (previously referred to as isolate EH202 [12] with a CCTCC No. M208068 and available upon request) was recently isolated from diseased turbot (*Scophthalmus maximus*) in a mariculture farm in Yantai, Shandong province of China and was routinely cultured on Luria-Bertani (LB) medium at 28°C. Genomic DNA was isolated from 10 ml overnight culture using the TIANamp Bacteria DNA Kit (TIANGEN Biotech, Beijing, China). Genomic DNA was quantified on 0.7% agarose gel stained with ethidium bromide and spectrophotometrically assessed. The stock DNA solution was separated into two aliquots, one for sequencing via pyrosequencing and the other stored at −80°C for further gap closing.

### High-density pyrosequencing and sequence assembly of the genome

The complete sequencing work was conducted using Roche GS FLX system [54]. A total of 286,550 reads counting up to 64,706,315 bases (averaged read length as 225 bp), were obtained resulting in a 17-fold coverage of the genome. Assembly was performed using the GS *de novo* Assembler software (http://www.454.com/) and produced 64 contigs ranging from 500 bp to 337,284 bp (the N50 contig size is 116,367 bp). Relationship of the contigs was determined by multiplex PCR [55]. Gaps were then filled in by sequencing the PCR products using ABI 3730xl capillary sequencers. Phred, Phrap and Consed software packages (http://www.genome.washington.edu) were used for the final assembly and edition, and low quality regions of the genome were resequenced. The assembly of the genome was verified by digestion of the genomic DNA with restriction enzymes and then running the products with pulsed-field gel electrophoresis (PFGE).

### Sequence analysis and annotation

Putative CDSs were identified by GeneMark [56] and Glimmer3 [57], and peptides shorter than 30 aa were eliminated. Sequences from the intergenic regions were compared to GenBank's non-redundant (nr) protein database [58] to identify genes missed by the Glimmer or GeneMark prediction and to detect pseudogenes. Insert sequences were first detected using IS Finder database (http://www-is.biotoul.fr/is.html) with default parameters and selected manually. Transfer RNA genes were predicted by tRNAScan-SE [59], while ribosomal DNAs (rDNAs) and other RNA genes were identified by comparing the genome sequence to the rRNA database [60], [61] and by using Infernal program [62]. Functional annotation of CDSs was performed through searching against nr protein database using BLASTP [63]. The protein set was also searched against COG (http://www.ncbi.nlm.nih.gov/COG/; [64]) and the KEGG (Kyoto encyclopedia of genes and genomes; http://www.genome.jp/kegg/) [65] for further function assignment. The criteria used to assign function to a CDS were (1) a minimum cutoff of 40% identity and 60% coverage of the protein length and (2) at least two best hits among the COG, KEGG, or nr protein database. A search for gene families in the genome was performed by BLASTCLUST. Subcellular localization of the proteins was predicted by PSORTb program (v2.0.1) [66]. TatP 1.0 server (v2.0) [67] and TATFIND 1.2 program [39] were used to detect the potential substrates of the Tat secretion system. Pathogenicity islands and anomalous genes were detected by PAI-IDA [68] and SIGI-HMM [69], respectively.

### Construction of phylogenetic tree

Phylogenetic position of *E. tarda* EIB202 within the *Enterobacteriaceae* was determined based on the protein sequences of 44 housekeeping genes (*adk*, *aroC*, *dnaA*, *dnaK*, *frr*, *fusA*, *gapA*, *gyrA*, *gryB*, *infC*, *nusA*, *pgk*, *phoB*, *phoR*, *pyrG*, *recC*, *rplA*, *rplB*, *rplC*, *rplD*, *rplE*, *rplF*, *rplK*, *rplL*, *rplM*, *rplN*, *rplP*, *rplS*, *rplT*, *rpmA*, *rpoA*, *rpoB*, *rpoC*, *rpoE*, *rpsB*, *rpsC*, *rpsE*, *rpsI*, *rpsJ*, *rpsK*, *rpsM*, *rpsS*, *smpB*, and *tsf*) [70]. BLAST algorithm was used when needed and ambiguous regions were trimmed according to an embedded mask. Concatenated protein sequences were aligned by ClustalW [71]. Maximum likelihood tree based on the aligned protein sequences was constructed by using PhyML [72] with 100 bootstrap iterations.

### Genome comparison

Orthologs between *E. tarda* EIB202 and other *Enterobacteriaceae* bacteria (*Escherichia coli* K-12 substr MG1655, *Erwinia carotovora* atrosepticum SCRI1043, *Klebsiella pneumoniae* subsp. pneumoniae MGH 78578, *Salmonella typhimurium* LT2, *Serratia proteamaculans* 568, *Shigella flexneri* 5 str. 8401, *Yersinia pestis* CO92, *Enterobacter sakazakii* ATCC BAA-894 and *Photorhabdus luminescens* subsp. laumondii TTO1) were detected by all-vs-all reciprocal_BLASTP search against the protein sets of these strains (http://www.ncbi.nlm.nih.gov/RefSeq), respectively. Criteria were as following: (1) E-value = e^−20^ or less and (2) >40% amino acid sequence identity, then the best hit was selected. Predicted *E. tarda* EIB202-specific genes were detected by screening EIB202 protein set against orthologs.

### Statistical analysis

The 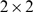 contingency Chi-square tests were performed to detect the significant differences between the counts of ORFs in each COG category for EIB202 and other *Enterobacteriaceae* bacterium (http://img.jgi.doe.gov). In the significant difference test,
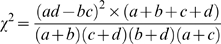
in which *a*, *b* were the observed numbers of ORFs in in each COG category for EIB202 and other *Enterobacteriaceae* bacterium, and *c*, *d* were the counts of the rest of all ORFs in each COG category for EIB202 and other *Enterobacteriaceae* bacterium, respectively. Significant differences were determined at *P*<0.05 (critical value 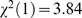) [73].

### Data Availability

The nucleotide sequence of the *E. tarda* EIB202 chromosome and the plasmid pEIB202 were submitted to the GenBank database under accession numbers CP001135 and CP001136, respectively.

## Supporting Information

Table S1 Genomic features of E. tarda EIB202 and other sequenced enterobacteria
(0.04 MB DOC)

Table S2 Amino acid biosynthesis genes in E. tarda EIB202
(0.17 MB DOC)

Table S3 The predicted Tat substrates in EIB202
(0.07 MB DOC)
