# Supplementary material for: Genome Sequence of the Versatile Fish Pathogen Edwardsiella tarda Provides Insights into its Adaptation to Broad Host Ranges and Intracellular Niches
Source: PLoS One. 2009 Oct 29;4(10):e7646. doi: 10.1371/journal.pone.0007646 (PMC2764856; doi:10.1371/journal.pone.0007646)
Supplement: Table S1 — Genomic features of E. tarda EIB202 and other sequenced enterobacteria (0.04 MB DOC) [file pone.0007646.s001.doc]

**Table S1. Genomic features of *E. tarda* EIB202 and other sequenced enterobacteria.**

| **Features** | **Bacteria** | | | | | | | | |
| --- | --- | --- | --- | --- | --- | --- | --- | --- | --- |
|  | EIB202 | *E. sakazakii* | *E. coli* k12 | *K. pneumoniae* | *P. luminescens* | *S. typhimurium* LT2 | *S. proteamaculans* | *S. flexneri* | *Y. pestis* |
| **Genome Size(bp)** | 3,760,463 | 4,530,777 | 4,639,675 | 5,694,894 | 5,688,987 | 4,951,371 | 5,495,657 | 4,574,284 | 4,829,855 |
| **Number of plasmids** | 1 | 2 | 0 | 5 | 0 | 1 | 1 | 0 | 3 |
| **GC content(%)** | 59.7 | 56.73 | 50.79 | 57.15 | 42.83 | 52.24 | 55.02 | 50.92 | 47.64 |
| **Protein-coding sequences** | 3,563 | 4,448 | 4,391 | 5,195 | 4,895 | 4,565 | 4,954 | 4,271 | 4,157 |
| **Average CDS size(bp)** | 909 | 899 | 918 | 927 | 941 | 916 | 957 | 890 | 931 |
| **Percent of coding region(%)** | 86.1 | 89.73 | 90.91 | 86.44 | 83.35 | 87.71 | 88.2 | 85.44 | 82.81 |
| **Ribsomal RNA genes** | 25 | 16 | 23 | 25 | 23 | 22 | 21 | 23 | 20 |
| **Number of tRNAs** | 95 | 82 | 85 | 86 | 84 | 86 | 85 | 96 | 69 |
