# Supplementary material for: Genome Sequence of the Versatile Fish Pathogen Edwardsiella tarda Provides Insights into its Adaptation to Broad Host Ranges and Intracellular Niches
Source: PLoS One. 2009 Oct 29;4(10):e7646. doi: 10.1371/journal.pone.0007646 (PMC2764856; doi:10.1371/journal.pone.0007646)
Supplement: Table S2 — Amino acid biosynthesis genes in E. tarda EIB202 (0.17 MB DOC) [file pone.0007646.s002.doc]

**Table S2. Amino acid biosynthesis genes in *E. tarda* EIB202**

| **Amino acid** | **CDS** | **Description** | **Species containing homologs** | **Identity** |
| --- | --- | --- | --- | --- |
| **Alanine** |  |  |  |  |
|  | **?** |  |  |  |
| **Arginine** |  |  |  |  |
|  | ETAE_3111 | Ornithine carbamoyltransferase | *Yersinia bercovieri* | 79% |
|  | ETAE_3479 | Argininosuccinate lyase | *Shigella sonnei* | 79% |
|  | ETAE_3480 | Argininosuccinate synthase | *Photorhabdus luminescens* | 82% |
| **Asparagine** |  |  |  |  |
|  | ETAE_2623 | Asparagine synthase (glutamine-hydrolysing) | *Erwinia carotovora* | 83% |
|  | ETAE_3521 | Aspartate--ammonia ligase | *Serratia proteamaculans* | 83% |
| **Aspartate** |  |  |  |  |
|  | ETAE_1487 | L-asparaginase | *Yersinia bercovieri* | 82% |
|  | ETAE_2179 | L-asparaginase | *Escherichia albertii* | 88% |
| **Cysteine** |  |  |  |  |
|  | ETAE_1134 | Cysteine synthase | *Sodalis glossinidius* | 74% |
|  | ETAE_3456 | Serine O-acetyltransferase | *Yersinia frederiksenii* | 86% |
| **Glutamate** |  |  |  |  |
|  | ETAE_3353 | Glutamate dehydrogenase (NADP+) | *Enterobacter sp.* | 83% |
| **Glutamine** |  |  |  |  |
|  | ETAE_3493 | Glutamine synthetase | *Serratia proteamaculans* | 94% |
| **Glycine** |  |  |  |  |
|  | ETAE_2821 | Glycine hydroxymethyltransferase | *Salmonella enterica* | 86% |
| **Histidine** |  |  |  |  |
|  | ETAE_0446 | Histidinol-phosphate aminotransferase | *Providencia stuartii* | 47% |
|  | ETAE_0792 | Aminoacylhistidine dipeptidase | *Yersinia mollaretii* | 72% |
|  | ETAE_1402 | Ribose-phosphate pyrophosphokinase | *Providencia stuartii* | 96% |
|  | ETAE_2275 | ATP phosphoribosyltransferase | *Serratia proteamaculans* | 90% |
|  | ETAE_2276 | Histidinol dehydrogenase | *Salmonella typhimurium* | 70% |
|  | ETAE_2277 | Histidinol-phosphate aminotransferase | *Escherichia coli* | 63% |
|  | ETAE_2278 | Imidazoleglycerol-phosphate dehydratase  / Histidinol-phosphatase | *Escherichia coli* | 74% |
|  | ETAE_2279 | Glutamine amidotransferase | *Erwinia tasmaniensis* | 72% |
|  | ETAE_2280 | Phosphoribosylformimino-5-aminoimidazole  carboxamide ribotide isomerase | *Serratia proteamaculans* | 69% |
|  | ETAE_2281 | Cyclase HisF | *Yersinia pseudotuberculosis* | 85% |
|  | ETAE_2282 | Phosphoribosyl-ATP pyrophosphohydrolase  / Phosphoribosyl-AMP cyclohydrolase | *Yersinia mollaretii* | 67% |
|  | ETAE_2801 | Histidyl-tRNA synthetase | *Salmonella enterica* | 84% |
| **Isoleucine** |  |  |  |  |
|  | ETAE_0088 | Acetolactate synthase I/II/III large subunit | *Serratia proteamaculans* | 75% |
|  | ETAE_0089 | Acetolactate synthase II small subunit | *Enterobacter sakazakii* | 68% |
|  | ETAE_0090 | Branched-chain amino acid aminotransferase | *Serratia proteamaculans* | 86% |
|  | ETAE_0091 | Dihydroxy-acid dehydratase | *Erwinia carotovora* | 80% |
|  | ETAE_0092 | Threonine dehydratase | *Yersinia intermedia* | 82% |
|  | ETAE_0094 | Ketol-acid reductoisomerase | *Salmonella enterica* | 83% |
|  | ETAE_0625 | Acetolactate synthase I/II/III large subunit | *Yersinia pestis* | 80% |
|  | ETAE_0626 | Acetolactate synthase I/III small subunit | *Photorhabdus luminescens* | 80% |
| **Leucine** |  |  |  |  |
|  | ETAE_0088 | Acetolactate synthase I/II/III large subunit | *Serratia proteamaculans* | 75% |
|  | ETAE_0091 | Dihydroxy-acid dehydratase | *Erwinia carotovora* | 80% |
|  | ETAE_0094 | Ketol-acid reductoisomerase | *Salmonella enterica* | 83% |
|  | ETAE_0618 | 3-Isopropylmalate/(R)-2-Methylmalate  dehydratase small subunit | *Salmonella enterica* | 73% |
|  | ETAE_0619 | 3-Isopropylmalate/(R)-2-Methylmalate  Dehydratase large subunit | *Yersinia bercovieri* | 77% |
|  | ETAE_0621 | 2-Isopropylmalate synthase | *Yersinia pestis* | 80% |
|  | ETAE_0625 | Acetolactate synthase I/II/III large subunit |  |  |
|  | ETAE_0626 | Acetolactate synthase I/III small subunit | *Photorhabdus luminescens* | 80% |
|  | ETAE_1982 | 3-Isopropylmalate/(R)-2-Methylmalate  dehydratase small subunit | *Thermosinus carboxydivorans* | 59% |
|  | ETAE_2033 | 3-Isopropylmalate/(R)-2-Methylmalate  dehydratase large subunit | *Thermosinus carboxydivorans* | 66% |
| **Lysine** |  |  |  |  |
|  | ETAE_0122 | Diaminopimelate epimerase | *Yersinia enterocolitica* | 76% |
|  | ETAE_0201 | Aspartate kinase | *Yersinia pseudotuberculosis* | 66% |
|  | ETAE_0340 | Lysyl-tRNA synthetase, class II | *Salmonella enterica* | 84% |
|  | ETAE_0565 | Aspartate kinase | *Yersinia intermedia* | 74% |
|  | ETAE_0591 | Dihydrodipicolinate reductase | *Salmonella enterica* | 77% |
|  | ETAE_0632 | UDP-N-acetylmuramoylalanyl-D-glutamate  --2,6-diaminopimelate ligase | *Yersinia mollaretii* | 68% |
|  | ETAE_0633 | UDP-N-acetylmuramoylalanyl-D-glutamyl-2,6  -diaminopimelate--D-alanyl-D-alanine ligase | *Erwinia carotovora* | 70% |
|  | ETAE_0734 | 2,3,4,5-Tetrahydropyridine-2-carboxylate  N-succinyltransferase | *Serratia proteamaculans* | 87% |
|  | ETAE_1096 | Dihydrodipicolinate synthase | *Serratia marcescens* | 76% |
|  | ETAE_1106 | Succinyl-diaminopimelate desuccinylase | *Erwinia carotovora* | 76% |
|  | ETAE_1972 | Diaminopimelate decarboxylase | *Vibrio splendidus* | 55% |
|  | ETAE_2416 | Aspartate-semialdehyde dehydrogenase | *Yersinia pestis* | 75% |
|  | ETAE_2911 | Diaminopimelate decarboxylase | *Serratia proteamaculans* | 72% |
|  | ETAE_2924 | Lysyl-tRNA synthetase, class II | *Yersinia bercovieri* | 84% |
|  | ETAE_3108 | Diaminopimelate decarboxylase | *Salmonella enterica* | 88% |
|  | ETAE_3247 | Acetylornithine/N-succinyldiaminopimelate  aminotransferase | *Serratia proteamaculans* | 72% |
|  | ETAE_3344 | Aspartate-semialdehyde dehydrogenase | *Serratia proteamaculans* | 83% |
|  | ETAE_3429 | Homoserine dehydrogenase | *Serratia proteamaculans* | 85% |
| **Methionine** |  |  |  |  |
|  | ETAE_0142 | 5-Methyltetrahydropteroyltriglutamate—  homocysteine methyltransferase | *Yersinia enterocolitica* | 69% |
|  | ETAE_0200 | 5-Methyltetrahydrofolate--homocysteine  methyltransferase | *Serratia proteamaculans* | 78% |
|  | ETAE_0201 | Aspartate kinase | *Yersinia pseudotuberculosis* | 66% |
|  | ETAE_0565 | Aspartate kinase | *Yersinia intermedia* | 74% |
|  | ETAE_1055 | Cystathionine beta-lyase | *Delftia acidovorans* | 59% |
|  | ETAE_2416 | Aspartate-semialdehyde dehydrogenase | *Yersinia pestis* | 75% |
|  | ETAE_3190 | Homoserine O-succinyltransferase | *Edwardsiella ictaluri* | 95% |
|  | ETAE_3429 | Homoserine dehydrogenase | *Serratia proteamaculans* | 85% |
|  | ETAE_3430 | Cystathionine gamma-synthase | *Yersinia bercovieri* | 77% |
| **Phenylalanine** |  |  |  |  |
|  | ETAE_2835 | Prephenate dehydratase | *Yersinia frederiksenii* | 73% |
|  | ETAE_3049 | Chorismate mutase | *Photorhabdus luminescens* | 60% |
|  | ETAE_3187 | Aromatic-amino-acid transaminase | *Serratia proteamaculans* | 64% |
| **Proline** |  |  |  |  |
|  | ETAE_0796 | Glutamate 5-kinase | *Yersinia intermedia* | 89% |
|  | ETAE_0797 | Glutamate-5-semialdehyde dehydrogenase | *Serratia proteamaculans* | 71% |
|  | ETAE_3400 | Pyrroline-5-carboxylate reductase | *Salmonella enterica* | 61% |
| **Serine** |  |  |  |  |
|  | ETAE_0539 | Phosphoserine phosphatase | *Yersinia mollaretii* | 72% |
|  | ETAE_2178 | Phosphoserine aminotransferase | *Erwinia carotovora* | 77% |
|  | ETAE_2949 | D-3-phosphoglycerate dehydrogenase | *Serratia proteamaculans* | 82% |
| **Threonine** |  |  |  |  |
|  | ETAE_2221 | Threonine aldolase | *Yersinia bercovieri* | 70% |
| **Tryptophan** |  |  |  |  |
|  | ETAE_1530 | Tryptophan synthase alpha chain | *Escherichia albertii* | 55% |
|  | ETAE_1531 | Tryptophan synthase beta chain | *Erwinia carotovora* | 84% |
|  | ETAE_1532 | Indole-3-glycerol phosphate synthase | *Yersinia enterocolitica* | 60% |
|  | ETAE_1533 | Anthranilate phosphoribosyltransferase | *Pectobacterium carotovorum* | 64% |
|  | ETAE_1534 | Anthranilate synthase component II | *Yersinia frederiksenii* | 74% |
|  | ETAE_1535 | Anthranilate synthase component I | *Yersinia bercovieri* | 67% |
| **Tyrosine** |  |  |  |  |
|  | ETAE_0446 | Histidinol-phosphate aminotransferase | *Providencia stuartii* | 47% |
|  | ETAE_2277 | Histidinol-phosphate aminotransferase | *Escherichia coli* | 63% |
|  | ETAE_2836 | Prephenate dehydrogenase | *Serratia proteamaculans* | 77% |
|  | ETAE_3049 | Chorismate mutase | *Photorhabdus luminescens* | 60% |
|  | ETAE_3187 | Aromatic-amino-acid transaminase | *Serratia proteamaculans* | 64% |
| **Valine** |  |  |  |  |
|  | ETAE_0088 | Acetolactate synthase I/II/III large subunit | *Serratia proteamaculans* | 75% |
|  | ETAE_0089 | Acetolactate synthase II small subunit | *Enterobacter sakazakii* | 68% |
|  | ETAE_0090 | Branched-chain amino acid aminotransferase | *Serratia proteamaculans* | 86% |
|  | ETAE_0091 | Dihydroxy-acid dehydratase | *Erwinia carotovora* | 80% |
|  | ETAE_0094 | Ketol-acid reductoisomerase | *Salmonella enterica* | 83% |
|  | ETAE_0625 | Acetolactate synthase I/II/III large subunit | *acetolactate synthase,* | 79% |
|  | ETAE_0626 | Acetolactate synthase I/III small subunit | *Photorhabdus luminescens* | 80% |
