# Supplementary material for: Genome Sequence of the Versatile Fish Pathogen Edwardsiella tarda Provides Insights into its Adaptation to Broad Host Ranges and Intracellular Niches
Source: PLoS One. 2009 Oct 29;4(10):e7646. doi: 10.1371/journal.pone.0007646 (PMC2764856; doi:10.1371/journal.pone.0007646)
Supplement: Table S3 — The predicted Tat substrates in EIB202 (0.07 MB DOC) [file pone.0007646.s003.doc]

**Table S3. The predicted Tat substrates in EIB202.**

| **Gene** | **Name** | **Putative function** | **Homologs**  **verified in**  ***E. coli*** | **Shared by other**  **Enterobacteriaceae**  **bacteria** | **Redox proteins** |
| --- | --- | --- | --- | --- | --- |
| ETAE_0096 | *rep* | ATP-dependent DNA helicase Rep |  |  |  |
| ETAE_0199 | *sufI* | Repressor protein for FtsI | Y | Y |  |
| ETAE_0224 | *citF* | Citrate lyase alpha chain/citrate-ACP transferase |  |  |  |
| ETAE_0225 | *citE* | Citrate lyase beta subunit |  |  |  |
| ETAE_0298 | *torA* | Trimethylamine-N-oxide reductase TorA | Y | Y | Y |
| ETAE_0446 |  | Hypothetical protein |  |  |  |
| ETAE_0478 |  | Hypothetical protein |  |  |  |
| ETAE_0714 | *amiC* | N-acetylmuramoyl-L-alanine amidase | Y | Y |  |
| ETAE_0798 |  | Putative NADP-dependent oxidoreductases |  | Y | Y |
| ETAE_0987 | *ampG* | Beta-lactamase induction signal transducer |  |  |  |
| ETAE_1116 | *napA* | Periplasmic nitrate reductase, large subunit |  | Y | Y |
| ETAE_1126 | *amiA* | N-acetylmuramoyl-L-alanine amidase | Y | Y |  |
| ETAE_1237 |  | Isochorismatase family protein |  |  |  |
| ETAE_1632 |  | Hypothetical protein |  |  |  |
| ETAE_1647 | *ttrB* | Tetrathionate reductase, subunit B |  |  | Y |
| ETAE_1649 |  | Putative tetrathionate reductase, subunit A |  |  | Y |
| ETAE_1741 |  | Putative collagenase |  |  |  |
| ETAE_1817 |  | Hypothetical protein |  |  |  |
| ETAE_1845 | *phsA* | Thiosulfate reductase precursor |  | Y | Y |
| ETAE_2156 |  | Hypothetical protein |  |  |  |
| ETAE_2195 | *dmsA* | Anaerobic dimethyl sulfoxide reductase subunit A | Y |  | Y |
| ETAE_2284 |  | Molybdopterin-containing oxidoreductase |  | Y |  |
| ETAE_2366 | *hybA* | Hydrogenase 2 protein |  | Y | Y |
| ETAE_2367 | *hybO* | Hydrogenase 2 small subunit | Y | Y | Y |
| ETAE_2494 |  | Hypothetical protein |  |  |  |
| ETAE_2793 | *nrfC* | Formate-dependent nitrite reductase; Fe-S centers |  | Y | Y |
| ETAE_2945 |  | Putative peptidase M24 |  | Y |  |
| ETAE_3142 |  | Oxidoreductase molybdopterin binding |  |  | Y |
| ETAE_3155 |  | Hypothetical protein |  |  |  |
| ETAE_3191 |  | Beta-lactamase |  |  |  |
| ETAE_3207 | *rplQ* | 50S ribosomal protein L17 |  | Y |  |
| ETAE_3223 | *rplP* | 50S ribosomal protein L16 |  | Y |  |
| ETAE_3337 |  | Formate dehydrogenase-O, major subunit |  | Y | Y |
